# Standardizing Competency and Bridging Implementation Gaps: Response to “Beyond Access: Should Competency Rather Than Confidence Drive Point-of-Care Ultrasound Integration in Internal Medicine Residency?”

**DOI:** 10.1016/j.ajmo.2026.100141

**Published:** 2026-08-12

**Authors:** Ugochukwu Ebubechukwu, Onyinye Ugoala, Chinenye Okafor, Festus Ibe, Duaa Mohamed, Anderson Anuforo, Ayesha Samad, Oboseh John Ogedegbe, Ahmad Abdelkhalek, Samy I. McFarlane, Pooja Belligund

**Affiliations:** a SUNY Downstate Health Sciences University, Brooklyn, NY; b Texas Tech Health Sciences University, Amarillo, Tex; c Medical University of South Carolina Regional Network, Florence, SC; d Jefferson Einstein Hospital, Philadelphia, Penn; e SUNY Upstate Medical University, Syracuse, NY; f Ocean University Medical Center, Brick, NJ; g Trinity Health, Ann Arbor, Mich; h Northwestern Medicine McHenry Hospital/Rosalind Franklin University, McHenry, Ill; i Veterans Affairs New York Harbor Healthcare System Brooklyn Campus, NY

The Reply,

We sincerely thank Drs Arun Babu and Sharmila for their thoughtful correspondence regarding our recent study examining point-of-care ultrasound (POCUS) utilization among internal medicine (IM) residents in the United States.^1^ We appreciate their thoughtful appraisal and agreement on the urgency of integrating structured POCUS education into internal medicine residency programs. The authors appropriately emphasize an important distinction between learner confidence and clinical competency, and we appreciate the opportunity to further clarify the intent and implications of our work. Their commentary raises important educational paradigms, specifically moving from self-reported confidence to objective competency, that warrant direct engagement as we collectively shape the future of medical education.

Our study was designed as a national needs assessment to characterize current patterns of POCUS utilization, identify perceived barriers, and explore residents' self-reported confidence across multiple POCUS applications.^1^ As noted in our manuscript, confidence should not be interpreted as a surrogate for competency, particularly given the well-known limitations of self-assessment and the potential influence of the Dunning-Kruger effect.^1^ We agree that competency-based assessment is the appropriate benchmark for evaluating educational success. However, assessing subjective confidence remains a crucial first step in national baseline assessments. POCUS confidence metrics highlight psychological barriers and comfort zones among trainees. Understanding where trainees lack confidence (eg, focused cardiac exams and e-FAST) enables program directors to design targeted, high-yield interventions. While objective tools, such as objective structured clinical examinations (OSCEs), represent the gold standard for clinical readiness, tracking trainee self-reported confidence provides crucial feedback on overall program culture and trainee readiness to engage in hands-on practice. Accordingly, our conclusions intentionally focused on educational gaps and implementation barriers rather than assertions regarding resident proficiency.

OSCEs, standardized image review, longitudinal quality assurance, entrustable professional activities, and direct observation are all valuable approaches for determining whether curricular interventions translate into improved clinical performance. However, before competency can be meaningfully assessed at scale, it is necessary to understand the educational environment in which learners train. Our survey sought to address this foundational question by identifying the structural and institutional barriers that currently limit opportunities for deliberate practice and supervised skill acquisition.

The authors note that relying on descriptive statistics limits isolating program-level variables (like curricula, faculty expertise, rotations) from post-graduate year seniority. While our nationwide snapshot serves as a foundational descriptive study, we agree that future multivariable modeling is needed. Identifying predictors of POCUS proficiency, such as machine access or ultrasound director presence, will clarify effective interventions. This study aimed to provide a national practice snapshot, not determine predictors of competency or confidence. The confidence increase with training should be seen as hypothesis generating. Future multicenter studies with objective assessments, standardized curricula, and multivariable analyses are vital for linking educational strategies to proficiency.

The authors highlight the gap between frequent procedural scans and low confidence in diagnostic use, like cardiac or lung exams. Our survey was designed to identify areas of current POCUS use, and we observed that procedural and IVC use stood out as frequent POCUS exams. We believe this correlates with the following: trainees have integrated IVC into their workflow, which likely stems from the volume assessment step in the Surviving Sepsis Bundle; and US-guided intravenous access remains largely assigned to trainees across the country.

Finally, we agree that curriculum standardization should extend beyond defining educational content to include competency benchmarks and robust assessment frameworks. Indeed, we believe these goals are complementary rather than competing. Standardized curricula establish consistent learning opportunities, whereas competency-based assessment ensures that these opportunities translate into safe, effective bedside practice. Both elements are essential for the successful integration of POCUS into IM training.

The next evolution in IM residency training should focus on translating curricular access into objective competency benchmarks, standardized EPA assessments, and patient-centered clinical outcomes. We appreciate the authors' insightful observations, which reinforce the need for the next generation of POCUS research to move beyond identifying barriers toward evaluating measurable educational and patient-centered outcomes. We hope our study serves as a foundation for this important work.

## CRediT authorship contribution statement

**Ugochukwu Ebubechukwu:** Conceptualization, Data curation, Formal analysis, Investigation, Methodology, Project administration, Visualization, Writing – original draft, Writing – review & editing. **Onyinye Ugoala:** Data curation, Methodology, Visualization, Writing – original draft. **Chinenye Okafor:** Data curation, Formal analysis, Methodology, Validation, Writing – original draft. **Festus Ibe:** Data curation, Methodology, Visualization, Writing – original draft. **Duaa Mohamed:** Data curation, Writing – original draft. **Anderson Anuforo:** Data curation, Writing – review & editing. **Ayesha Samad:** Data curation, Writing – review & editing. **Oboseh John Ogedegbe:** Data curation, Writing – review & editing. **Ahmad Abdelkhalek:** Data curation, Writing – review & editing. **Samy I. McFarlane:** Project administration, Resources, Supervision, Writing – review & editing. **Pooja Belligund:** Conceptualization, Investigation, Project administration, Writing – original draft, Writing – review & editing.

## Declaration of Competing Interest

None.
